# Prolonging herd immunity to cholera via vaccination: Accounting for human mobility and waning vaccine effects

**DOI:** 10.1371/journal.pntd.0006257

**Published:** 2018-02-28

**Authors:** Corey M. Peak, Amanda L. Reilly, Andrew S. Azman, Caroline O. Buckee

**Affiliations:** 1 Center for Communicable Disease Dynamics, Department of Epidemiology, Harvard T.H. Chan School of Public Health, Boston, Massachusetts, United States of America; 2 Department of Applied Mathematics, John A. Paulson School of Engineering and Applied Sciences, Harvard University, Cambridge, Massachusetts, United States of America; 3 Department of Epidemiology, Johns Hopkins Bloomberg School of Public Health, Baltimore, Maryland, United States of America; International Vaccine Institute, REPUBLIC OF KOREA

## Abstract

**Background:**

Oral cholera vaccination is an approach to preventing outbreaks in at-risk settings and controlling cholera in endemic settings. However, vaccine-derived herd immunity may be short-lived due to interactions between human mobility and imperfect or waning vaccine efficacy. As the supply and utilization of oral cholera vaccines grows, critical questions related to herd immunity are emerging, including: who should be targeted; when should revaccination be performed; and why have cholera outbreaks occurred in recently vaccinated populations?

**Methods and findings:**

We use mathematical models to simulate routine and mass oral cholera vaccination in populations with varying degrees of migration, transmission intensity, and vaccine coverage. We show that migration and waning vaccine efficacy strongly influence the duration of herd immunity while birth and death rates have relatively minimal impacts. As compared to either periodic mass vaccination or routine vaccination alone, a community could be protected longer by a blended “Mass and Maintain” strategy. We show that vaccination may be best targeted at populations with intermediate degrees of mobility as compared to communities with very high or very low population turnover. Using a case study of an internally displaced person camp in South Sudan which underwent high-coverage mass vaccination in 2014 and 2015, we show that waning vaccine direct effects and high population turnover rendered the camp over 80% susceptible at the time of the cholera outbreak beginning in October 2016.

**Conclusions:**

Oral cholera vaccines can be powerful tools for quickly protecting a population for a period of time that depends critically on vaccine coverage, vaccine efficacy over time, and the rate of population turnover through human mobility. Due to waning herd immunity, epidemics in vaccinated communities are possible but become less likely through complementary interventions or data-driven revaccination strategies.

## Author summary

Cholera vaccination can be a relatively quick means to temporarily prevent cholera from spreading in an at-risk population. In order to understand how long this temporary protection remains and therefore the timeline for when we need to install longer-term water and sanitation solutions, we must know how long we can expect the vaccine to provide herd protection. To answer this and other related questions, we developed a mathematical model to test different vaccination strategies in a simulated population and in a case study of a displaced-persons camp in Bentiu, South Sudan. We found that the duration of vaccine-derived herd protection can be short (<1 year) in settings of moderate transmission potential and high population mobility, but this duration can be extended through a strategy that complements a one-time mass vaccination campaign with ongoing, routine vaccination. We show that short-lived vaccine efficacy and high population turnover in the Bentiu camp can help explain why the camp had a cholera outbreak despite two high-coverage vaccination campaigns in the two previous years. Our results support, and provide timelines for, cholera vaccination as initial protection while longer-term structural interventions can be implemented.

## Introduction

Vaccination campaigns with sufficiently high efficacy and coverage can ideally achieve herd immunity in the population. Herd immunity emerges when the indirect protection of vaccination reduces the per-case expected number of onward infections in a population (i.e., the effective reproductive number, R_e_) below one—whether these onward infections are direct person-to-person or indirect environmental transmission pathways.[1–3] While the balance between these direct and indirect pathways will depend on the setting, the role of both pathways had been described for cholera[4] and utilized in modeling literature.[5–7] Herd immunity is not permanent, however, and is expected to wane over time via short-lived vaccine efficacy and an influx of susceptible, unvaccinated individuals. Due to a reliable efficacy profile and high attainable coverage, killed oral cholera vaccines (kOCV) can generate powerful herd protection effects.[8,9] In 2012, the World Health Organization (WHO) created a kOCV stockpile to facilitate vaccine usage in three settings: (1) humanitarian crises at high risk of cholera importation and transmission; (2) high-endemicity “hot spots”; and (3) cholera outbreaks.[10] As the stockpile approaches its fifth year, evaluation of its management must address uncertainties in sustainability and long-term strategy, particularly regarding the duration of herd immunity (DHI) in these three settings.

Regarding the first setting, kOCVs can be a quick stopgap measure to protect cholera-prone dynamic populations such as refugee camps,[11] but it remains unclear how much time is “bought” by vaccination before longer-term solutions such as water, sanitation, and hygiene promotion are necessary. Second, feasibility and economic analyses of vaccination in endemic “hot spot” settings are strongly influenced by the frequency of revaccination.[12] Third, it remains to be seen how strongly, and in what direction, population mobility should be considered when prioritizing target populations for reactive vaccination during outbreaks.

These are not merely hypothetical concerns. Beginning in October 2016, the Bentiu Protection of Civilians (PoC) Camp in South Sudan sustained a cholera outbreak despite high-coverage two-dose mass vaccination campaigns in both 2014 and 2015 which were intended to preemptively stave-off the risk of cholera.[13,14] Consequently, questions have emerged about the utility of vaccination and the expected risk of outbreaks,[15] particularly in dynamic populations where cholera often breaks out.[16] Modeling studies of other diseases (e.g., [17–21]) suggest a suite of factors which may have contributed to the camp’s susceptibility to an outbreak, including waning vaccine efficacy, the influx of susceptible displaced people, an extremely high birth rate, and resettlement of vaccinated individuals. However, the relative contributions of these factors and their implications for vaccination strategy in the future are not clear.

Here we examine the implications of vaccine waning and human mobility on herd immunity over time in non-endemic settings, providing new insights related to the risk of outbreaks in vaccinated populations. Using mathematical models, we compare how well several common vaccination strategies sustain herd immunity and we demonstrate the non-monotonic relationship between migration rate and the projected impact of pre-emptive vaccination. We analyze data from the Bentiu PoC Camp to quantify the impact of expected drivers of waning herd immunity and assess whether they are sufficient to explain the vulnerability of the camp to the observed outbreak.

## Methods

### Model

**Fig 1:**
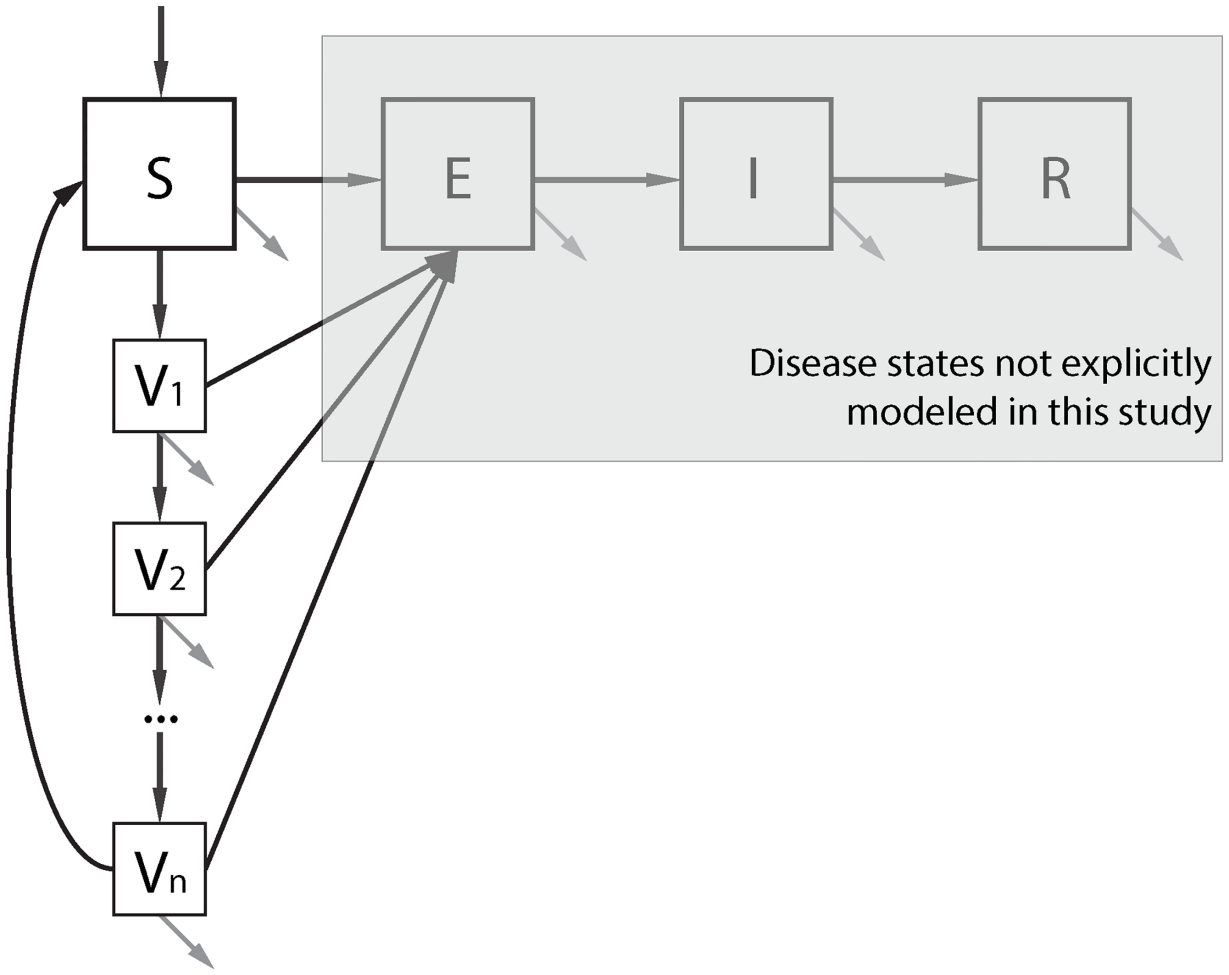
Mathematical model framework. Susceptible individuals (*S*) can become vaccinated (*V*_1_) and proceed through each monthly vaccine compartments (*V*_1_,*V*_2_, …,*V*_*n*_). Individuals enter the system through birth and immigration (top arrow) and leave the system through death and emigration (grey arrows). The force of infection for individuals in a compartment *V*_*i*_ is reduced by a factor of 1 − *VE*(*i*) according to a leaky model of vaccine action. For reference, traditional disease progression compartments for exposed but not yet infectious (E), infectious (I), and recovered (R) are shown, but are not explicitly modeled due to the focus of this study on vaccine-derived herd immunity.

We developed a system of deterministic differential equations to model a well-mixed population that is being targeted with vaccination. The population compartments of principal interest for this study are individuals who are fully susceptible to disease, *S*, and those who were vaccinated *n*-months ago, *V*_*n*_ (Fig 1). To account for the observation that kOCV direct effects do not tend to wane exponentially,[22,23] we created an ensemble of *n* monthly stages (*V*_1_,*V*_2_, …,*V*_*n*_), which collectively generate an Erlang-distribution for the duration of time in the *V*-ensemble.[24,25] We set the mean time residing in any *V*_*n*_ compartment to 30.5 days; therefore, susceptible individuals move after vaccination to compartment *V*_1_ for an average of one month, then to *V*_2_ for an average of one month, and so forth. See supporting materials for the system of differential equations (S1 Text).

The system of ordinary differential equations was solved using the *deSolve* package[26] in the statistical software program R (version 3.2.4). All code used to generate this paper can be freely found at https://github.com/peakcm/cholera.

### Vaccination strategies

Vaccination is implemented according to two approaches: mass and routine. We model mass vaccination as a large fraction of individuals moving into the *V*_1_ compartment on a particular day, possibly recurrently (e.g., annually). Routine vaccination moves a substantially smaller fraction of individuals into the *V*_1_ compartment for many days in a row. In each approach, vaccine priority is given first to susceptible individuals, *S*, then those who were vaccinated the longest time ago (i.e., *V*_*n*_, then *V*_*n*−1_, and so on until reaching the allotted number of vaccines for that day). In addition to mass vaccination and routine vaccination, we test a blended “Mass and Maintain” strategy in which one-time mass vaccination is followed by routine vaccination. See supplemental materials for mathematical details on modeling mass vaccination transition rates (S1 Text).

Currently, a complete kOCV course includes two doses administered approximately two weeks apart.[10] However, because the timescale of interest for this study is measured in years, not days, we assume mass vaccination campaigns elapse over one day and provide protection instantaneously. Furthermore, for generalizability across disease systems, we focus on the number of vaccine courses rather than the number of actual vaccines.

We parameterized the time-varying vaccine efficacy, *VE*(*t*), of kOCV (whole-cell with B-subunit) using estimates from a large clinical trial in Bangladesh (see Discussion for more details on choice of vaccine).[22,27] Recent meta-analysis results found no differences in two-dose efficacy for vaccines with and without the B-subunit.[23] To provide monthly estimates of vaccine efficacy, *VE*(*t*), we used published 6-month point estimates with linear interpolations between each. Efficacy after the 5^th^ year is assumed to be zero, as the estimated mean efficacy becomes negative.[22] Therefore, our last *V*_*n*_ compartment before returning to full susceptibility is at 60 months (*V*_60_).

### Human mobility

We assume individuals emigrate from the population at a rate that is the same regardless of vaccination status. The total population size, *N*(*t*), is held constant by offsetting emigration with an equal rate of immigration, unless otherwise noted. Our main results assume that incoming migrants bring neither vaccine-derived nor naturally-acquired immunity into the population.

We estimated migration rates from three example settings where kOCVs have been used, including: (1) a ‘stable’ urban population; (2) a highly mobile urban population; and (3) a displaced person setting with intermediate mobility. First, to represent a stable urban population, we estimate a migration rate of $\frac{1}{20\;years}$ (i.e., an average residence time of 20 years) from the observation that only 9% of an OCV study population in Calcutta, India, changed in the two years following vaccination in 2006.[28] Secondly, to represent a highly mobile urban population, we estimate a migration rate of $\frac{1}{2\;years}$ from the observation that 58% of a study population in Dhaka, Bangladesh, had relocated over two years.[29] Thirdly, to represent a displacement camp with intermediate mobility, we estimate a resettlement rate of $\frac{1}{4.3\;years}$ in the Bentiu PoC Camp in South Sudan in the period from February to October 2016, during which the International Organization on Migration (IOM) reports a rather stable population of 104,000 people and approximately 2,000 monthly individuals both entering the camp and resettling from the camp (S1 Fig) [http://www.iomsouthsudan.org/tracking/].

### Outcome measurements

We define the duration of herd immunity (DHI) as the amount of time following a vaccination campaign with an effective reproductive number, *R*_*e*_, below one. We calculate

$$R_{e}(t)\;=\;X(t)*R_{0}$$ (1)

where *X*(*t*) is the proportion of the population susceptible at time *t*,

$$X(t)\;=\;\frac{S(t)+\sum_{i\;=\;1}^{n}V_{i}(t)(1-VE(i))}{N(t)}.$$ (2)

Our modeling framework serves to estimate the key proportion *X*(*t*) dynamically. From this value, we derive *R*_*e*_(*t*), DHI, and the probability of an outbreak as follows.

Due to the special behavior of deterministic models, we perform the following adjustments. When a simulation asymptotically approaches *R*_*e*_ (*t*) = 1 from below, we define DHI as the time until *R*_*e*_ (*t*) ≥ 0.99. Because epidemic extinction is possible in reality when *R*_0_ > 1 (and, conversely, epidemic propagation is possible when *R*_0_ < 1), we use our calculation of *R*_*e*_ (*t*) to estimate the probability of an outbreak. When *R*_*e*_ > 1, the final epidemic size tends to follow a bimodal distribution with a probability of sporadic die-out and a probability of a large epidemic. Using a recent method for computing epidemic final size distributions,[30] we find the threshold of 10 cases is a reasonable cutoff size such that a large outbreak is henceforth very likely for sizeable values of *R*_*e*_ (S2 Fig). We therefore define an outbreak as more than 10 cases and, by assuming a Poisson distribution of secondary infections (mean = *R*_*e*_), we can calculate the probability of an outbreak of more than *y* cases initiated by a single infectious case using the Borel-Tanner distribution:[31,32]

$$Pr(Y>y)\;=\;1-\sum_{i\;=\;1}^{y}\frac{1}{(i-1)!}i^{i-2}{R_{e}}^{i-1}e^{-iR_{e}}.$$ (3)

### Mobility-informed vaccination targeting

To assess the role of mobility on the optimal pre-emptive targeting of kOCVs, we simulate a setting with migration rates ranging from zero, representing a closed population, to a very high value of $\frac{1}{1\;year}$ (i.e., an average residence time of one year). Since we focus here on an at-risk population in a non-endemic setting, our outcome of interest is the cumulative probability (C) of sustaining a cholera outbreak that was seeded by an imported case, which equals one minus the probability of having no outbreaks greater than *y* cases:

$$C\;=\;1-\prod_{t\;=\;1}^{D}\left({\left(1-Pr(Y>y)\right)}^{I_{mig}}\right)$$ (4)

where D is the duration of follow-up time in days, *y* is the minimum outbreak size, and *I*_*mig*_ *is* the expected number of infected individuals who migrate into the population in one day. *I*_*mig*_ is calculated by:

$$I_{mig}\;=\;\pi N(e^{m}-1),$$ (5)

where *π* is the probability an incoming migrant is infected, *N* is the size of the targeted population, *m* is the daily migration rate, and therefore the daily number of incoming migrants equals *N*(*e*^*mt*^ − 1) where *t* = 1 day. We assume each imported case has an independent probability of starting an outbreak of more than *y* cases given the effective reproductive number *R*_*e*_(*t*) on that day *t*.

We measure the difference between the cumulative outbreak probability, C, over *D* days in the absence of vaccination as compared to the first *D* days following mass vaccination. A larger difference suggests a more impactful vaccination intervention. For our main results, we focus on a setting with moderate transmissibility (*R*_0_ = 1.5)[6,33] and set the probability that a migrant is infected, *π*, equal to $\frac{1}{N}$, which simplifies Eq 5 to *I*_*mig*_ = (*e*^*mt*^ − 1) (S1 Text).

### Bentiu PoC Camp case study

We examine the suspected drivers of waning herd immunity in a well-described outbreak in the Bentiu PoC Camp in South Sudan. Of the three million persons targeted for health resources in broader South Sudan, including the Bentiu PoC Camp, UNFPA expects 335 deliveries per day, which equates to birth rate of approximately $\frac{1}{24.4\;years}$.[34] We assumed this to be our demographic turnover rate as a conservatively high estimate.

We estimated population susceptibility over time, *X*(*t*), in six scenarios (Table 1). In the “observed” scenario, we used empirical measures of four key drivers of waning herd immunity, specifically: the birth/death rate of $\frac{1}{24.4\;years}$; an empirical distribution of efficacy over time, *VE*(*t*); a camp resettlement rate of $\frac{1}{4.3\;years}$ (i.e., an average camp residence time of 4.3 years) which is balanced by an equal rate of entries for a net-zero impact on *N*(*t*); and a dynamic population size, *N*(*t*), driven by net growth or shrinkage through camp entries or exits. We compare this scenario with counterfactual scenarios that eliminate at least one of these drivers and will therefore increase DHI. We constructed a composite counterfactual scenario in which: the birth/death rate was set to zero; vaccine efficacy was held constant at its maximum value (70.3%) for all time since vaccination; the camp resettlement rate was set to zero; and the population size was held constant at approximately the level observed during the outbreak (100,000). To isolate the impact of each driver of waning herd immunity, we ran simulations where one driver is set to the “observed” condition while the other three drivers are set to their counterfactual condition to remove their influence (Table 1).

**Table 1:** Relative contribution of four potential drivers of waning herd immunity in Bentiu PoC Camp.

Scenario	Vaccine Efficacy *VE*(*t*)	Population Size *N*(*t*)	Birth & Death Rate	Resettle-ment Rate	Percent Susceptible on Oct 16, 2016* X(*t*)	Difference in Percent Susceptible Δ*X*(*t*)	Attributable Percent
**Composite Counterfactual**	70.3%	100,000	0	0	34.4%	--	--
**Only *VE*(*t*) waning**	Empirical	100,000	0	0	58.2%	23.8%	34.9%
**Only *N*(*t*) changes**	70.3%	Empirical	0	0	56.6%	22.2%	32.6%
**Only Births & Deaths**	70.3%	100,000	$\frac{1}{24.4\;years}$	0	38.1%	3.7%	5.4%
**Only Resettlement**	70.3%	100,000	0	$\frac{1}{4.3\;years}$	52.9%	18.5%	27.1%
**‘Observed’**	Empirical	Empirical	$\frac{1}{24.4\;years}$	$\frac{1}{4.3\;years}$	80.8%	46.3%	**--**

* October 16, 2016 is the date of the first cholera case reported from the outbreak in the Bentiu PoC Camp

To assess the relative importance of each driver of waning herd immunity in this case study, we calculate a measure of attributable percent. For a scenario *i* that isolates one driver, we measure the proportion susceptible (*X*(*t*)_*i*_) on October 16, 2016, the start of the observed outbreak. To compare scenarios, we calculate the difference between estimates of the proportion susceptible at the start of the outbreak under scenario *i* with estimates in the composite counterfactual scenario,

$${\Delta X(t)}_{i}\;=\;{X(t)}_{i}-{X(t)}_{composite}.$$ (6)

Finally, we calculate the percent of waning herd immunity attributable to each driver (*AR*%),

$$AR\%\;=\;100*{\Delta X(t)}_{i}/\sum {\Delta X(t)}_{i}.$$ (7)

In order to estimate the probability of an outbreak given introduction of a cholera case using the population susceptibility over time, *X*(*t*), we must estimate the basic reproductive number, *R*_0_. Following frameworks[35,36] recently applied to cholera in South Sudan,[37] we retrospectively estimate the time-varying reproductive number using two sources: (1) daily case reports, which we extract from Cholera Situation Reports from the South Sudan Ministry of Health,[38] and (2) an expected generation interval distribution, which we assume to follow a discretized gamma distribution with median of 5 days.[37] This method assumes uniform mixing, no imported cases after the first case, and no missing data.[35,36] Maximum likelihood estimation procedures were implemented in the statistical software program R using the *R0* package.[39]

## Results

### Dynamics of population susceptibility and herd immunity

**Fig 2:**
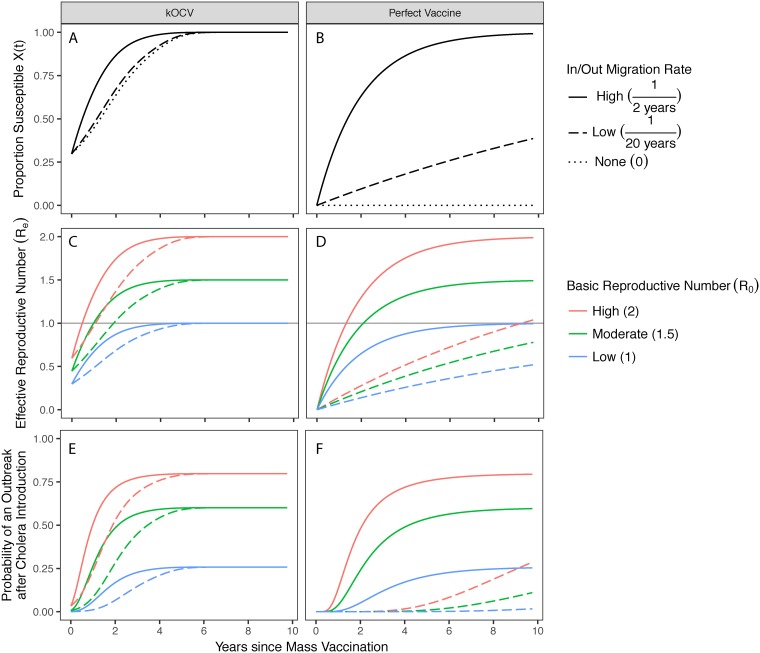
Dynamics of population susceptibility and herd immunity. Dynamics following mass vaccination (100% coverage) with kOCV (left column) or a hypothetical vaccine with VE = 1 indefinitely (right column). (**A-B**) Population susceptibility increases over time in the presence of migration rates of $\frac{1}{2\;years}$ (solid line), $\frac{1}{20\;years}$ (dashed line), and zero (dotted). (**C-D**) The effective reproductive number changes over time with X(t) differently for settings with basic reproductive numbers of 2 (red), 1.5 (green), and 1 (blue). (**E-F**) The probability that a single case sparks an outbreak of more than 10 cases. Birth and death rates are set to zero in each simulation.

Following mass vaccination with 100% coverage, population susceptibility, *X*(*t*), quickly increases over time in the presence of high migration rates and short-lived vaccine efficacy (Fig 2A, solid line). Even with a hypothetical perfect vaccine that retains complete protection indefinitely, high migration rates can drive population susceptibility near 100% within 9–10 years (Fig 2B, solid line). Between three primary drivers causing herd immunity to wane, namely migration, waning efficacy, and demographic turnover through births and deaths, we find that the first two are substantially more influential than either the birth or death rate, which are each typically much slower processes. As compared to rates of birth and death set to zero, even pessimistic estimates of a life expectancy of 40 years result in negligible differences in the proportion of the population susceptible due to the relatively faster rates of other drivers (S3 Fig).

Following kOCV vaccination with 100% coverage in a population with high migration, we estimate the vaccine-derived DHI to be approximately 0.47 years when *R*_0_ = 2, 0.98 years when *R*_0_ = 1.5, and 4.06 years when *R*_0_ = 1 (Fig 2C, solid lines). These durations increase to 1.07 years, 1.89 years, and 5.16 years, respectively, in the presence of low migration rates instead (Fig 2C, dashed lines). As expected, DHI is reduced when vaccine coverage is less than 100%, and, depending on both the coverage and *R*_0_, herd immunity is sometimes unattainable (S4 Fig).

Achieving herd immunity is a key theoretical threshold, but in reality an outbreak is possible below the threshold and is not guaranteed above the threshold.[40] Mass vaccination reduces, but does not eliminate, the probability that an imported case sparks an outbreak for a duration of time that depends critically on the migration rate and how vaccine efficacy wanes over time (Fig 2E and 2F). For example, even though herd immunity is lost within just 0.47 years in a high migration setting when *R*_0_ = 2 (Fig 2C, solid red line), the outbreak probability is kept below 50% for twice as long (Fig 2E, solid red line).

### Optimizing revaccination with “Mass and Maintain” strategies

**Fig 3:**
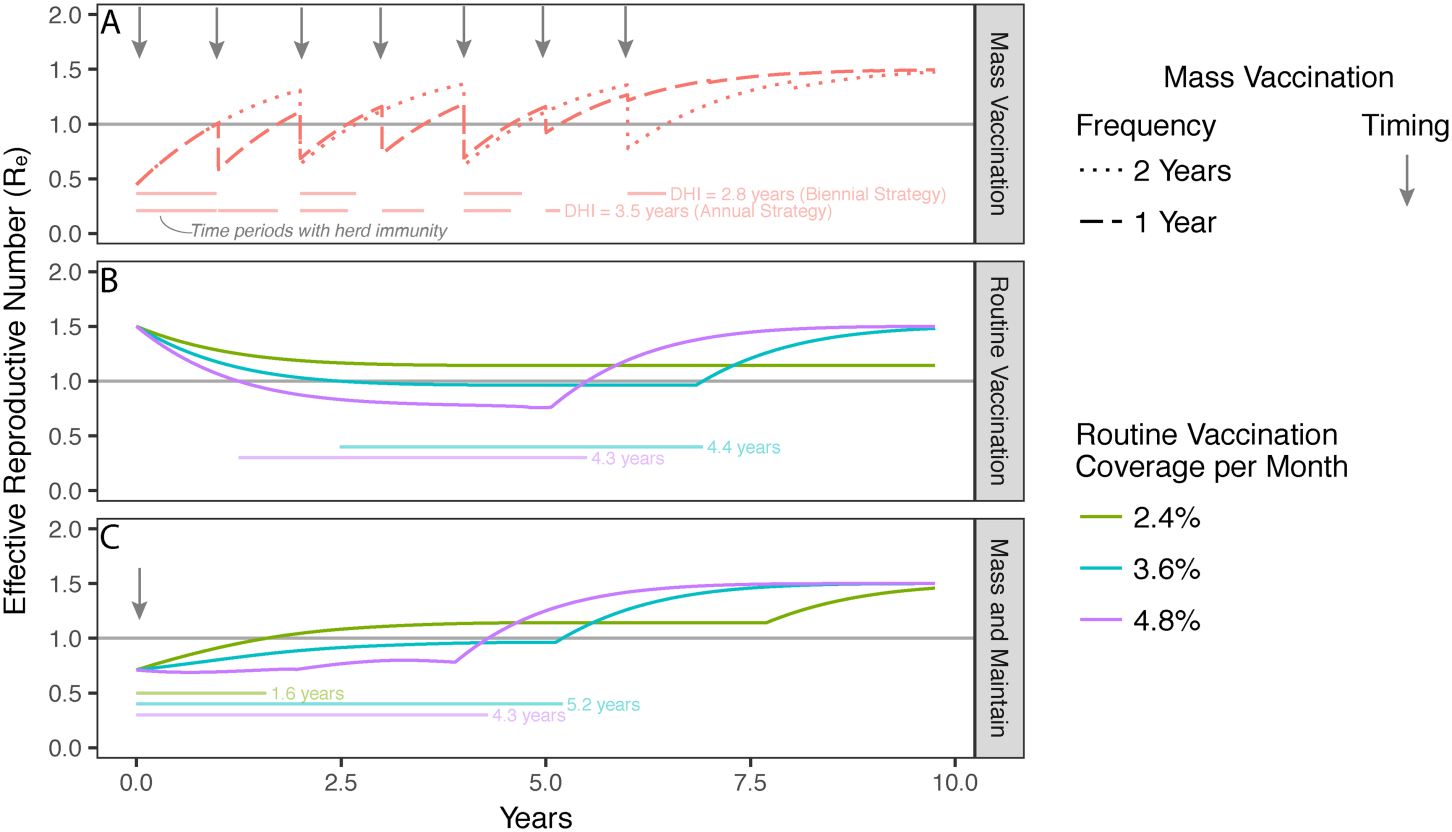
Revaccination strategies to maximize duration of herd immunity (DHI). (**A**) Recurring mass vaccination events (arrows) with 100% coverage of susceptible people every year (dashed line) or two years (dotted line) is shown to periodically achieve then lose herd immunity, designated by the horizontal line at *R*_*e*_ = 1. Faded horizontal bars show times with herd immunity under each strategy and the total DHI is annotated to the right of each. (**B**) Routine vaccination of 2.4% (green), 3.6% (teal), or 4.8% (purple) of the population per month achieve herd immunity for 0, 4.4, and 4.3 years, respectively. (**C**) A “Mass and Maintain” strategy with one-time vaccination at 75% coverage followed by routine vaccination of 2.4% (green), 3.6% (teal), or 4.8% (purple) of the population per month can render herd immunity for 1.6, 5.2, and 4.3 years, respectively. The following are held constant for all simulations: population size = 10,000; maximum vaccine courses = 30,000; *R*_0_ = 1.5; migration rate = $\frac{1}{2\;years}$; and birth and death rates = $\frac{1}{40\;years}$.

We considered several operational strategies for sustaining herd immunity through vaccination alone. In a hypothetical population of size *N* with *R*_0_ = 1.5 and a high rate of migration ($\frac{1}{2\;years}$), mass vaccination every year or every two years with 100% coverage of susceptible individuals can render herd immunity for 3.5 or 2.8 years, respectively, before depleting a fixed vaccine allotment of 3*N* full vaccine courses (Fig 3A). If these vaccines are instead allotted on a daily basis through routine vaccination, DHI can be extended to 4.4 years (Fig 3B). However, recurring mass campaigns have diminishing returns per vaccine once herd immunity is achieved; meanwhile routine vaccination alone requires a long period of time to build up herd immunity. We therefore find that a blended “Mass and Maintain” strategy that complements a single mass vaccination campaign with subsequent routine vaccination can maintain herd immunity longer than either strategy alone (Fig 3C), both for this example and for a wide range of settings with various migration rates and *R*_0_ values (S1 Table).

### Optimizing pre-emptive mass vaccination by targeting intermediate mobility settings

**Fig 4:**
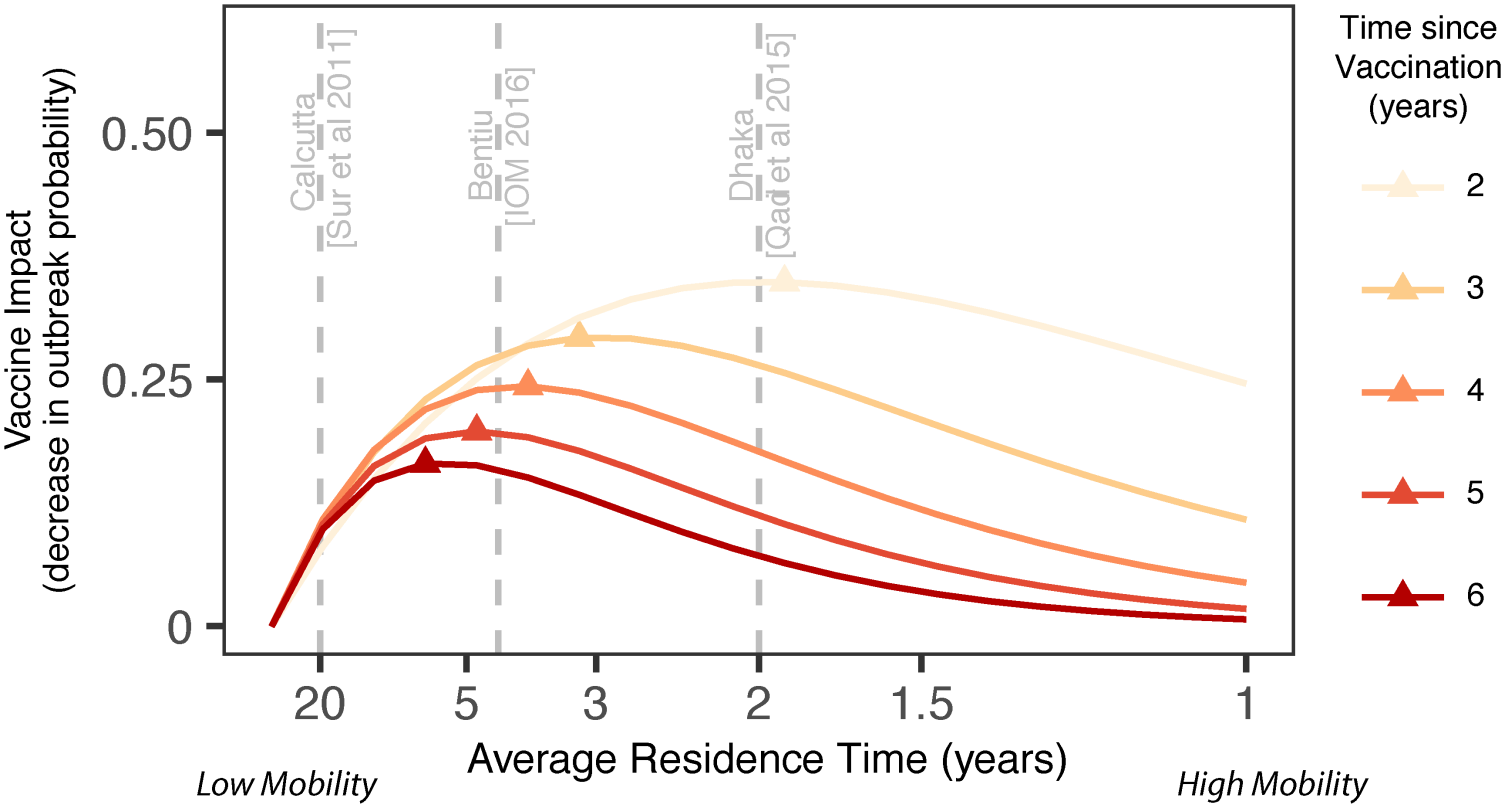
Vaccine targeting optimized in settings with intermediate rates of migration. Vaccine impact, as measured by the difference in the cumulative probability of an outbreak comparing a mass kOCV campaign (coverage 100%) versus no vaccination, is shown to reach maxima (triangles) at intermediate levels of mobility (x axis). The time since vaccination (colored lines) modifies these maxima. Grey dashed lines denote the estimated migration rates for Calcutta, Bentiu PoC Camp, and Dhaka. In this example, *R*_0_ = 1.5 and the average probability that a migrant is infected is 1/*N*, where *N* is the population size.

In addition to the importance of migration on DHI, one may posit that communities with higher migration rates are also more likely to have cholera imported. In order to optimize pre-emptive kOCV impact in at-risk settings, there is a tradeoff between targeting low-mobility communities, where herd immunity may last for a long time but cholera introduction is rare, and high-mobility communities, where the opposite is expected. We find that communities with intermediate levels of migration may experience the largest vaccine-derived decrease in outbreak risk sparked by an imported case (Fig 4). For example, the migration rate recorded in the Bentiu PoC Camp in mid-2016 is near the optimal condition for maximizing the impact of a single mass vaccination campaign in the 4–6 year time horizon, assuming *R*_0_ = 1.5. If one is more interested in shorter time horizons since vaccination, the migration rate that maximizes vaccine impact favors mobile communities, similar to some urban areas in Dhaka, Bangladesh.[29] Sensitivity analyses suggest that intermediate mobility rates (e.g., between those observed in Dhaka and Calcutta) generally maximize vaccine impact, but the optimal migration rate is slower in settings that have a larger population size, a higher transmission potential (*R*_0_), or where a higher fraction of incoming migrants are infected (e.g., due to high-burden neighbors) (S5 Fig). Conversely, settings with small population size, low transmission potential, and whose migrants have a small probability of being infectious require very high migration rates in order to garner much baseline risk of cholera importation and outbreak.

### Bentiu PoC Camp case study

**Fig 5:**
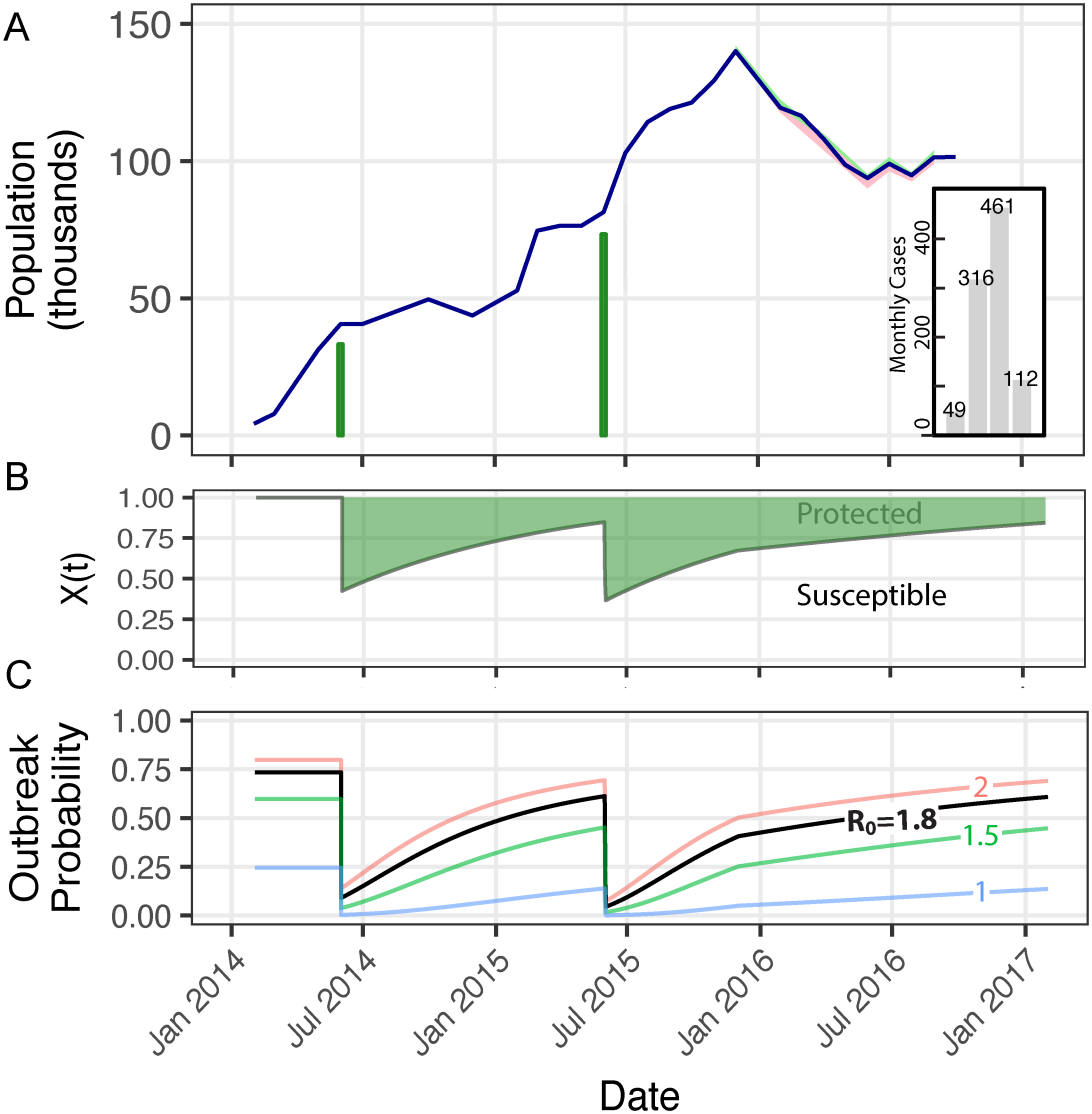
Bentiu PoC Camp case study. (**A**) Reported population size of the Bentiu PoC Camp (blue line), approximate number of people vaccinated assuming two-dose coverage (green bars), and monthly case counts from October to January (inset grey bars). IOM began reporting entries and exits in December 2015, which are represented by the faint green and red ribbons around the blue line. (**B**) The proportion susceptible over time (green line) decreases due to mass vaccination events and increases over time since vaccination. (**C**) The probability that a single case sparks an outbreak of more than 10 cases increases with *X*(*t*) and R_0_, as represented by line color: R_0_ = 1 (blue); 1.5 (green); 1.8 (black); and 2 (red). For reference, R_e_ = 0 yields an outbreak probability of 0; R_e_ = 1.01 yields a probability of 0.25; R_e_ = 1.35 yields a probability of 0.50; R_e_ = 1.84 yields a probability of 0.75; and R_e_>4.66 yields an outbreak probability over 99%.

The Bentiu PoC Camp grew from 4,291 occupants in February 2014 to a peak of 140,101 in December 2015 and then converged to approximately 104,000 in May 2016 (Fig 5A). Assuming a cholera-naïve population before vaccination, we estimate that only 37% of the camp remained susceptible after the second round of vaccination in June 2015. By the time that the first cholera case in the camp was detected (i.e., October 16, 2016), the camp susceptibility percentage increased to 81% (Fig 5B). By December 1, 2016, we estimate that only 40.5% of camp residents had ever been vaccinated, which closely matches a WHO/IOM survey performed that month that reported kOCV coverage of 40%.[38]

Using case reports and assuming a fixed generation interval distribution, we estimate the mean effective reproductive number, *R*_*e*_ (*t*), exceeded unity for approximately two months following the first case, with a maximum likelihood estimate of 1.45 (1.18–1.75) (S6 Fig). Using the population fraction susceptible of 0.81 estimated above, we calculate a basic reproductive number, *R*_0_, of approximately 1.80 in this setting in the absence of vaccination (Eq 1). These findings are within the range of estimates derived from South Sudan in 2014.[37] Assuming this pre-vaccination estimate of *R*_0_ = 1.80, we find that after vaccination the probability of an outbreak first exceeded 50% in May 2016 and reached 57% when the outbreak began in October (Fig 5C, black line). Using a “Mass and Maintain” strategy including vaccination of 100% of individuals migrating into the camp after the second mass vaccination campaign, we estimate that only 52% of the population would have been susceptible on the date the first case was reported in the camp, which is low enough to generate herd immunity at the time (assuming *R*_0_ = 1.80)(S7 Fig).

The drivers of waning herd immunity in this population, from strongest to weakest, were short-lived vaccine efficacy, population growth, camp resettlement rate, and demographic turnover via births and deaths (Table 1, S2 Table). In the counterfactual scenario lacking these drivers, we would expect that as few as 34% of the population were susceptible on the day of the first reported cases in Bentiu PoC, which would render herd immunity even if *R*_0_ was as high as 3.

## Discussion

Vaccination can rapidly protect a population at risk of a cholera outbreak, but the duration of vaccine-derived herd immunity depends critically on vaccine coverage, waning vaccine efficacy, and a net influx of susceptible people through population mobility. In our case study of the Bentiu PoC Camp, we find that quantification of these drivers help explain the vulnerability of this population to an outbreak despite two recent high-coverage vaccine campaigns. Therefore, disease re-emergence does not necessarily imply vaccine failure and can be avoided by data-driven revaccination strategies or by scaling-up long-term, broad-spectrum solutions while under the temporary cover of pathogen-specific vaccination. Our results provide key time windows during a population can expect to resist a cholera outbreak even if the pathogen were to be introduced.

One practical implementation of the “Mass and Maintain” vaccination strategy in a camp setting can include a one-time mass vaccination campaign followed by routine vaccination of new members of the population, such as births and new entries. Population sub-groups with high vulnerability and mobility, such as coastal fishing communities,[41] may also benefit from the “Mass and Maintain” vaccination strategy targeted at seasonal influxes of migrants such as new fishermen. In an urban or open population, such as Dhaka or Calcutta, routine identification of new members becomes more challenging, but performance of the WHO Expanded Programme on Immunization in cholera endemic regions like Bangladesh are promising.[42] Recent work has also shown serological triggers for periodic mass vaccination can be an effective alternative method to maintain herd immunity to measles.[43]. For cholera specifically, there is a need for more research into cross-sectional markers of immunity which can inform risk profiling, revaccination timing, and, if stratified by age, the impact of mass vaccination.[44] A growing area of research focuses on the vaccine efficacy, and duration of protection, provided by a single kOCV dose.[5,45] Such work will help elucidate the relative merits of revaccination versus ongoing vaccination of new arrivals, for example.

Current guidelines for the optimal use of the kOCV stockpile recommend targeting “areas with important population movements.”[46] Mobility is recognized as an important driver of the performance of vaccination strategies to control ongoing cholera outbreaks.[47] Here, we focus on pre-emptive vaccination of at-risk communities to show the competing effects of high mobility on expected vaccine impact. In order to operationalize the finding that vaccination may be most impactful for populations with intermediate degrees of mobility, data on migration rates from sources such as censuses or mobile phone call data records must be collected to define “intermediate” mobility for a given context.[48]

Long-term solutions to cholera, and many other waterborne diseases, include investments in water, sanitation, and hygiene infrastructure. In high-income countries where such systems are established, the reproductive number is expected to be far below unity and therefore the effective integration of new migrants, even from poorer or cholera-prone regions, is not expected to decrease herd protection of the host population. However, in areas such as informal settlements and large low-income cities, incoming migrants can put stress on an already fragile water and sanitation infrastructure, potentially pushing the reproductive number above one and rendering the population at risk of sustained cholera transmission.

Our results depend on several simplifying assumptions. By modeling a well-mixed population, we are assuming no heterogeneity in contact patterns or local reproductive numbers. In reality, we expect diseases, especially those like cholera with environmental transmission dynamics, to exhibit substantial spatial heterogeneity in transmission intensity.[49] These differences become crucial if, as we may expect, migration occurs at higher rates into sub-regions with higher transmission potential due to confounders like poverty and temporary housing. Critically, we would expect DHI to decrease, the probability of an outbreak to increase, and the routine vaccination of migrants to become even more crucial.

Our model assumes a leaky mode of vaccine action, whereby vaccination reduces the disease susceptibility of each recipient. Our calculation of proportion susceptible, *X*(*t*), is robust to other assumptions regarding the method by which vaccine effects wane, namely: time-dependent failure in “take,” corresponding to an all-or-nothing response; and time-dependent failure in “degree,” corresponding to a leaky vaccine response (S8 Fig).[50] Our parameterization of a waning leaky vaccine aligns with prevailing interpretations[22] of the clinical trial data,[27] but alternative explanations for changes in vaccine efficacy over time in a clinical trial are difficult to rule-out, such as frailty, loss to follow up, and random variability.[51]

The migration rates estimated from Dhaka, Bentiu, and Calcutta are intended for benchmarking purposes and do not imply that migration rates are either constant or generalizable to the whole city or region. Indeed, we would expect to retain herd immunity longer after vaccination for a given migration rate if the rate was calculated in a population which included a stable sub-group of permanent residents and a small, highly mobile sub-group of temporary residents.

Cholera vaccine efficacy has been shown to vary by age of recipient,[22,23,27] however for simplicity and lack of detailed data we do not model this age structure. If children respond poorly to kOCV and are members of a mass vaccination campaign, we would expect herd immunity to wane more quickly, and especially so if children are disproportionate sources of transmission. Furthermore, over the course of an outbreak, we may expect the relative contributions of different age groups to differ, which can have important consequences on vaccine impact and targeting.[52] Currently, little is understood about immunity to cholera, though the waning individual immunity could be derived from the central role of mucosal phenomena.[53] For simplicity, we focus on pre-emptive vaccination of a generalized population without previous exposure to cholera. Although kOCV with the B-subunit is less preferred for vaccine stockpile applications, our primary results present the kOCV efficacy profile with the B-subunit due to the biological plausibility of the estimates in the time-varying analysis[22] and the recent observation that two-dose vaccine efficacy with and without the B-subunit is likely indistinguishable.[23]

Though in reality many other forces likely contributed to the Bentiu PoC Camp’s susceptibility to the observed cholera outbreak, our case study shows that known key drivers (namely waning vaccine efficacy, a net influx of susceptible people through population mobility) alone are strong enough to produce the observation that the camp population sustained a cholera outbreak despite recent vaccination campaigns. Provided additional data on the cholera outbreak and the camp population, a complete cholera model fit to the epidemic may yield additional insights.

The model we present is not limited to cholera or other diseases with only short-duration or leaky vaccines (e.g., the typhoid capsular polysaccharide vaccine [54]). The phenomenon of waning herd immunity also has strong implications on disease control strategies that include mass vaccination or “mop up” vaccination, such as measles[55] and yellow fever.[56] For yellow fever in particular, fractional vaccine doses have been used to extend vaccine supply under the assumption that vaccine efficacy of fractional doses lasts at least one year.[57] Following the mass vaccination of 25 million people in Angola and the Democratic Republic of the Congo, routine vaccination may be the most efficient way to henceforth sustain herd immunity in these populations, should this be the goal. Human mobility and waning herd immunity are key considerations for when these urban populations should be revaccinated.

Herd immunity is a key target for the control of vaccine-preventable diseases and can be monitored over time using information on the vaccine efficacy and population turnover rates. We show this information is essential for optimizing revaccination strategies, targeting vaccine stockpiles, and explaining re-emergence of outbreaks in recently vaccinated populations.

## Supporting information

S1 Text Contents include the system of differential equations, vaccination transition rate calculation, vaccination targeting in intermediate mobility settings, and details on the interactive online supplement. (DOCX)

S1 Table Sensitivity analysis of revaccination strategy optimization. (DOCX)

S2 Table Magnitude of potential drivers of waning herd immunity in Bentiu PoC Camp using Backward Selection. (DOCX)

S3 Table Review of attack rates in select large recent epidemics. (DOCX)

S1 FigBentiu PoC Camp population estimates over time.In order to simulate the Bentiu PoC Camp, we separated the IOM population estimates (black line) into four segments (separated by vertical dashed lines). During the first segment from February 2014 to June 2014, we assumed linear population growth (blue line). During the second segment from June 2014 to December 2015, we simulated exponential growth at a rate of $\frac{1}{1.21\;years}$. During the third segment from December 2015 to May 2015, we assumed exponential decay at a rate of $\frac{1}{1.21\;years}$. During the fourth and final segment beginning May 2015, we assumed population size was constant. The use of exponential and constant segments allowed for population size to change dynamically within a compartmental model framework, and provided population estimates that were visually reasonable. Our model simulations began on June 15, when vaccination first occurred.(TIF)

S2 Fig Final epidemic size distribution and choice of cutoff. The final epidemic size distribution in a population of 1000 is monotonically decreasing when *R*_0_ equals 0.9 (red) and follows a bimodal distribution when *R*_0_ equals 1.2 (yellow), 1.5 (green), 1.8 (blue), and 2.5 (black). The inset shows a cutoff of 10 cases can discriminate large and small outbreaks with high sensitivity, but specificity can be low with low values of *R*_0_.
(TIF)

S3 FigChanges in the proportion of the population susceptible (*X*(*t*)) as a function of years since vaccination in the presence of non-zero birth rates.As per Fig 2A and 2B, but with the addition of high birth/death rates ($\frac{1}{40\;years}$) and the Whole Cell vaccine profile (without the B-subunit). Even conservatively fast rates of birth and death ($\frac{1}{40\;years})$ are slow compared to the rates of vaccine efficacy waning and high ($\frac{1}{2\;years})$ or low ($\frac{1}{20\;years}$) migration, and therefore have little impact. Note that linetype (i.e., solid, dashed, and dotted) apply to birth/death rates both high (black) and low (grey); therefore, dashed grey lines refer to simulations with no demographic turnover and low migration rates.(TIF)

S4 Fig Duration of herd immunity (DHI) as a function of vaccine coverage and basic reproductive number. For both the whole cell and whole cell (with B-subunit) kOCVs, DHI is maximized in settings with high vaccine coverage and low basic reproductive numbers. Migration rates are set to zero. Uncolored regions never obtain herd immunity.
(TIF)

S5 FigVaccine targeting sensitivity to *R*_0_ and the fraction of migrants infected, *μ*.With increases in *R*_0_ or the fraction of migrants infected, *μ*, the optimal migration rate decreases from the fastest tested rate, $\frac{1}{1\;year}$ (red), to the slowest tested rate, $\frac{1}{40\;years}$ (blue). Contour lines denote average residence time in years from case studies in Dhaka (2), Bentiu (4.3), and Calcutta (20). For each simulation, the population size, *N*, is set to 10,000.(TIF)

S6 Fig Time-dependent reproductive number (*R*_*t*_) and daily cholera case counts in Bentiu PoC Camp between October, 2016, and January, 2017. Using the daily case counts (grey bars) and a generation interval with median of 5 days and following a gamma distribution with shape = 0.5 and rate = 0.1 as per ref [37], we report a mean time-dependent reproductive number (red line) above unity for nearly two months. 95% confidence intervals are shown in pink.
(TIF)

S7 Fig Time-dependent proportion susceptibility, X(t), in the Bentiu PoC Camp in the presence of a Mass and Maintain vaccination strategy. As per Fig 5B, with an additional dashed line indicating a counterfactual scenario whereby vaccines were administered to 100% of the estimated 55,628 new entries to the camp after the second mass vaccination campaign. With this strategy, population susceptibility on October 16, 2016 is 0.52 (dashed line), as compared to 0.81 in the absence of the Mass and Maintain strategy (solid green line).
(TIF)

S8 Fig Calculation of *X*(*t*) is robust to vaccine efficacy waning due to time-dependent failures in “take” or “degree.” Vaccine efficacy waning that is due to a time-dependent failure in “take” (i.e., an “All or Nothing” vaccine waning) (left panel) retains a constant *VE*(*t*) (dashed lines) while the number of individuals in the *V*(*t*) ensemble decreases over time (dotted lines) from 75% to 0% using a theoretical example vaccine. For a time-dependent failure in “degree (i.e., a leaky vaccine waning) (right panel), individuals remain in the *V*(*t*) ensemble, but vaccine efficacy wanes from 75% to 0%. The proportion susceptible over time, *X*(*t*), calculated by Eq 5 is identical for both modes of action (solid lines).
(TIF)

S9 Fig Demonstration of logarithmic adjustment for transition rates. As the desired fraction of individuals to be vaccinated in a single day increases (x axis), the vaccination transition rate with the logarithmic adjustment (see supplementary materials) moves the accurate fraction of the population into the *V*_1_ compartment (solid line) while a transition rate that is simply equal to just the number of vaccines to be used (dashed line) does not move enough individuals into *V*_1_.
(TIF)
