# Supplementary material for: Prolonging herd immunity to cholera via vaccination: Accounting for human mobility and waning vaccine effects
Source: PLoS Negl Trop Dis. 2018 Feb 28;12(2):e0006257. doi: 10.1371/journal.pntd.0006257 (PMC5847240; doi:10.1371/journal.pntd.0006257)
Supplement: S1 Text — (DOCX) [file pntd.0006257.s001.docx]

**S1 Text**

*System of Differential Equations*

In this paper, we focus on the dynamics of vaccine-derived herd immunity and therefore can simplify a standard SEIRV model framework to the S (Susceptible) and V (Vaccinated) compartments (Fig 1). In order to accommodate vaccine protection that wanes non-linearly, we utilize an ensemble of *n* monthly stages ($V_{1}, V_{2},\ldots,V_{n}$), where *n* equals 60 for a vaccine with five-years of protection.

$$\frac{dS}{dt}=bN-dS-v_{s}(t)+\tau V_{n}+m_{i}N-m_{e}S$$

$$\frac{dV_{1}}{dt}= v_{s}(t)-\tau V_{1}-dV_{1}-m_{e}V_{1}$$

$$\frac{dV_{2}}{dt}= \tau V_{1}-\tau V_{2}-dV_{2}-m_{e}V_{2}$$

$$\ldots$$

$$\frac{dV_{n}}{dt}= \tau V_{n-1}-\tau V_{n}-dV_{n}-m_{e}V_{n}$$

where:

$N$ is the total population size ($N=S+V_{1}+V_{2}+\ldots+V_{n}$)

$b$ is the daily per-population birth rate

$d$ is the daily per-population death rate

$v_{s}(t)$ is the vaccination transition rate on day $t$ as defined in the next section of the supplemental text

$\tau$ is the transition rate between vaccination compartments, typically equal to $\frac{1}{30.5 days}$

$m_{i}$ is the immigration rate into the population system

$m_{e}$ is the emigration rate out of the population system

*Vaccination Transition Rate Calculation*

In the simplest case whereby vaccination occurs at the onset of the study, we initialize the model with compartment $V_{1}$ equal to the number of vaccine recipients and subtract these from the $S$ compartment. This approach suffices for one-time mass vaccination, but we must explicitly model vaccination transition rates for recurrent mass vaccination and routine vaccination. Assuming $n_{V}(t)$ vaccines are available for use on a given day, the vaccination transition rate from $S$ to $V_{1}$ on day $t$, $\nu_{S}(t)$, is calculated by:

$$\nu_{S}(t)=S*-\ln\left( 1-\min\left( 0.99,\frac{n_{V}(t)}{S} \right) \right).$$

When the number of vaccines allocated on a given day is much smaller than the number of susceptible individuals eligible to receive vaccination (e.g., $\frac{n_{V}(t)}{S}<0.1$), then the logarithmic adjustment term $-\ln\left( 1-\min\left( 0.99,\frac{n_{V}(t)}{S} \right) \right)$ will approach $\frac{n_{V}(t)}{S}$ and therefore the transition rate $\nu_{S}(t)$ will approximately equal the number of vaccine courses available (i.e., $\nu_{S}(t)\approx n_{V}(t)$) (Fig S9, dashed line).

However, when a substantial fraction of the population is to receive mass vaccination on a single day (e.g., $n_{V}(t)>0.1*S$), the number of vaccine courses available, $n_{V}(t)$, increasingly becomes a poor estimate for the transition rate, $\nu_{S}(t)$, needed to move the appropriate number of individuals into $V_{1}$. Therefore, the logarithmic adjustment term $-\ln\left( 1-\min\left( 0.99,\frac{n_{V}(t)}{S} \right) \right)$ inflates the transition rate and allows the deterministic solver to move the desired number of individuals into $V_{1}$ (Fig S9, solid line). For computational tractability, we assume the vaccine campaign coverage, $\frac{n_{V}(t)}{S}$, does not exceed 99%.

When the number of available vaccines, $n_{V}(t)$, exceeds the number of individuals in the $S$ compartment, then vaccines are then given first to those who were vaccinated the longest time ago (i.e., $V_{48}$ first, then $V_{47}$, and so on).

*Vaccination Targeting in Intermediate Mobility Settings*

In order to calculate the expected impact of vaccination on reducing the probability of an outbreak sparked by an imported case, we must assume individuals migrating into the population are infected with cholera with a certain probability ($\pi$). As described in Methods and Materials, we set $\pi$equal to $\frac{1}{N}$ for our main results, which simplifies Equation 5 to $I_{mig}=\left( e^{m}-1 \right)$ For main text Figure 4, we consider a population of size 10,000 and therefore $\pi=\frac{1}{10,000}=0.0001$. This value for $\pi$ is consistent with a rough estimate of the average fraction of the population infectious on a given day during large recent epidemics in Zimbabwe (2008-2009) and Haiti (2010-2015) (Table S3).

We present a sensitivity analysis of the optimal migration rate with values of $\pi$ between 0.00001 and 0.001 (an order of magnitude larger and smaller than the main result presented) and $R_{0}$ ranging from 0.75 to 3. We find when R_0_ and $\pi$ are large, representing a setting that has high transmission potential and a high influx of migration from cholera-affected neighbors, the impact of vaccination over a 4-year time horizon is optimized when migration is slow (i.e., the average residence time increases) (Fig S5, blue). Conversely, high migration rates will tend to optimize the impact of vaccination in settings where transmission potential is low and a small proportion of migrants are infected (Fig S5, red). Because the population size, $N$, and the fraction of migrants infected, $\pi$, are coupled in Equation 5, the variables behave similarly and therefore the optimal migration rate tends to decrease as the population size increases.

*Interactive Online Supplement*

The interactive online supplement can be found at: <https://coreypeak.shinyapps.io/herd_protection_estimator/>. To account for seasonal forcing, the transmission parameter ($\beta$) is allowed to vary with each day (t) according to a sinusoidal function $\beta\left( t \right)= 1+f*\cos(\tau+{2\pi*t}/{365})$ where *f* is the magnitude of seasonal forcing and $\tau$ is a frameshift parameter accounting for the time of initial vaccination campaign (τ=0 if vaccination occurs at the peak transmission season, τ=π if vaccination occurs at the trough of transmission season). Therefore, we assume an annual cycle, but note that some regions such as Dhaka may exhibit biannual cycles.[52]
