# Supplementary material for: Prolonging herd immunity to cholera via vaccination: Accounting for human mobility and waning vaccine effects
Source: PLoS Negl Trop Dis. 2018 Feb 28;12(2):e0006257. doi: 10.1371/journal.pntd.0006257 (PMC5847240; doi:10.1371/journal.pntd.0006257)
Supplement: S1 Table — (DOCX) [file pntd.0006257.s002.docx]

**Table S1. Sensitivity analysis of revaccination strategy optimization**

| R_0_ | Migration Rate  (years^-1^) | Optimal Strategy | DHI  (years) |
| --- | --- | --- | --- |
| 1.25 | 0 | Mass and Maintain | 18.3 |
| 1.50 | 0 | Mass and Maintain | 10.0 |
| 2.00 | 0 | Mass and Maintain | 5.1 |
| 2.50 | 0 | Mass and Maintain | 3.0 |
| 1.25 | $1/{20}$ | Mass and Maintain | 17.9 |
| 1.50 | $1/{20}$ | Mass and Maintain | 8.8 |
| 2.00 | $1/{20}$ | Mass and Maintain | 4.8 |
| 2.50 | $1/{20}$ | Mass and Maintain | 3.2 |
| 1.25 | $1/{4.3}$ | Mass and Maintain | 12.2 |
| 1.50 | $1/{4.3}$ | Mass and Maintain | 5.8 |
| 2.00 | $1/{4.3}$ | Mass and Maintain | 4.1 |
| 2.50 | $1/{4.3}$ | Mass and Maintain | 2.6 |
| 1.25 | $1/2$ | Mass and Maintain | 9.1 |
| 1.50 | $1/2$ | Mass and Maintain | 4.7 |
| 2.00 | $1/2$ | Mass and Maintain | 3.5 |
| 2.50 | $1/2$ | Mass and Maintain | 1.4 |
| 1.25 | $1/1$ | Mass and Maintain | 4.8 |
| 1.50 | $1/1$ | Mass and Maintain | 2.2 |
| 2.00 | $1/1$ | Mass and Maintain | 1.5 |
| 2.50 | $1/1$ | Mass and Maintain | 1.3 |

Comparison of the performance of Routine, Mass, and Mass and Maintain vaccination strategies with respect to DHI in a population of size 10,000, a vaccine supply of 30,000 courses, and the following operational parameters: routine vaccination of between 2 and 16 courses per day; mass vaccination coverage between 0 and 100% of susceptible individuals; and mass vaccination frequency between annual and every 3 years.
