# Supplementary material for: Prolonging herd immunity to cholera via vaccination: Accounting for human mobility and waning vaccine effects
Source: PLoS Negl Trop Dis. 2018 Feb 28;12(2):e0006257. doi: 10.1371/journal.pntd.0006257 (PMC5847240; doi:10.1371/journal.pntd.0006257)
Supplement: S2 Table — (DOCX) [file pntd.0006257.s003.docx]

**Table S2. Magnitude of potential drivers of waning herd immunity in Bentiu PoC Camp using Backward Selection.**

| **Scenario** | **Vaccine Efficacy**  $\boldsymbol{VE(t)}$ | **Population Size**  $\boldsymbol{N(t)}$ | **Birth &**  **Death Rate** | **Resettle-ment Rate** | **Percent Susceptible on Oct 16, 2016**  $\boldsymbol{X(t)}$ |
| --- | --- | --- | --- | --- | --- |
| **Composite Counterfactual** | 70.3% | 100,000 | 0 | 0 | 34.4% |
| **Remove Only** $\boldsymbol{VE(}\boldsymbol{t)}$ **waning** | 70.3% | Empirical | $\frac{1}{24.4 years}$ | $\frac{1}{4.3 years}$ | 70.5% |
| **Remove Only** $\boldsymbol{N(t)}$ **changes** | Empirical | 100,000 | $\frac{1}{24.4 years}$ | $\frac{1}{4.3 years}$ | 71.8% |
| **Remove Only Births & Deaths** | Empirical | Empirical | 0 | $\frac{1}{4.3 years}$ | 79.6% |
| **Remove Only Resettlement** | Empirical | Empirical | $\frac{1}{24.4 years}$ | 0 | 73.3% |
| **Observed** | Empirical | Empirical | $\frac{1}{24.4 years}$ | $\frac{1}{4.3 years}$ | 80.8% |

Backward removal of drivers reveals the same order of influential drivers as the forward selection of drivers (Table 1). In order of decreasing importance, these are vaccine efficacy, population size, resettlement, and births/deaths.
