# Supplementary material for: Prolonging herd immunity to cholera via vaccination: Accounting for human mobility and waning vaccine effects
Source: PLoS Negl Trop Dis. 2018 Feb 28;12(2):e0006257. doi: 10.1371/journal.pntd.0006257 (PMC5847240; doi:10.1371/journal.pntd.0006257)
Supplement: S3 Table — (DOCX) [file pntd.0006257.s004.docx]

**Table S3. Review of attack rates in select large recent epidemics.**

| Country | Year | Cases Reported^1^ | Population^2^  (thousands) | Attack Rate  (per thousand) | | Daily Proportion Infectious^3^ |
| --- | --- | --- | --- | --- | --- | --- |
|  |  |  |  | Annual | Weekly Average |  |
| Zimbabwe | 2008 | 60,055 | 13,495 | 4.45 | 0.09 | 0.00005 |
|  | 2009 | 68,151 | 13,721 | 4.97 | 0.10 | 0.00005 |
| Haiti | 2010 | 179,379 | 10,000 | 17.93 | 0.34 | 0.00017 |
|  | 2011 | 340,311 | 10,145 | 33.54 | 0.65 | 0.00033 |
|  | 2012 | 112,076 | 10,289 | 10.89 | 0.21 | 0.00011 |
|  | 2013 | 58,809 | 10,431 | 5.64 | 0.11 | 0.00006 |
|  | 2014 | 27,753 | 10,572 | 2.62 | 0.05 | 0.00003 |
|  | 2015 | 36,045 | 10,711 | 3.37 | 0.06 | 0.00003 |
| Crude Average | | | | 10.43 | 0.20 | 0.00010 |

^1^ http://gamapserver.who.int/gho/interactive_charts/cholera/atlas.html

^2^ <https://esa.un.org/unpd/wpp/>

^3^ Assuming an average duration of infectiousness of 3.5 days,[51] half the weekly average cases would be infectious on a given day.
