# Supplementary figures and images for: Prolonging herd immunity to cholera via vaccination: Accounting for human mobility and waning vaccine effects
Source: PLoS Negl Trop Dis. 2018 Feb 28;12(2):e0006257. doi: 10.1371/journal.pntd.0006257 (PMC5847240; doi:10.1371/journal.pntd.0006257)

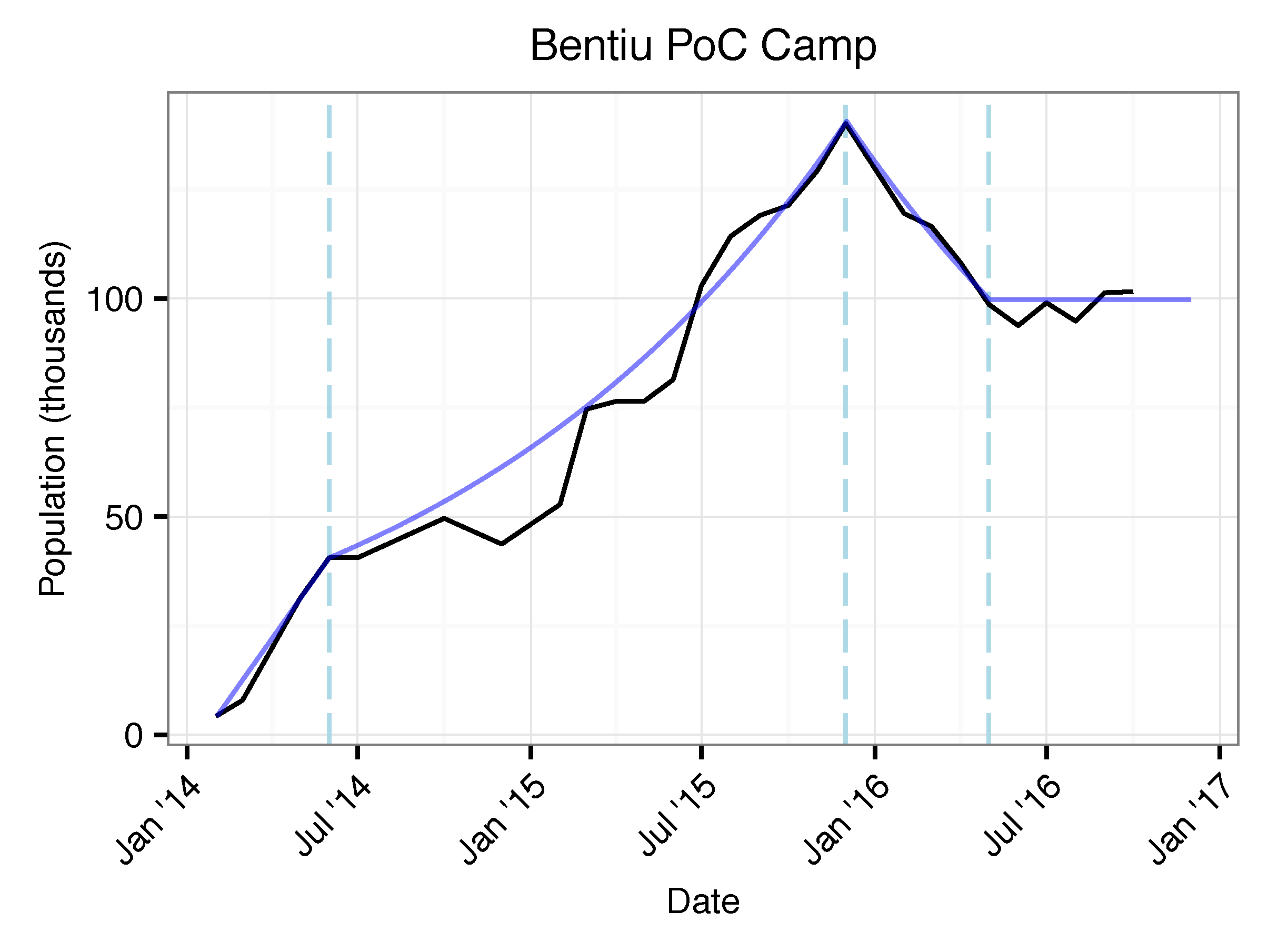

Supplement: S1 Fig — In order to simulate the Bentiu PoC Camp, we separated the IOM population estimates (black line) into four segments (separated by vertical dashed lines). During the first segment from February 2014 to June 2014, we assumed linear population growth (blue line). During the second segment from June 2014 to December 2015, we simulated exponential growth at a rate of 11.21years. During the third segment from December 2015 to May 2015, we assumed exponential decay at a rate of 11.21years. During the fourth and final segment beginning May 2015, we assumed population size was constant. The use of exponential and constant segments allowed for population size to change dynamically within a compartmental model framework, and provided population estimates that were visually reasonable. Our model simulations began on June 15, when vaccination first occurred. (TIF) [file pntd.0006257.s005.tif]

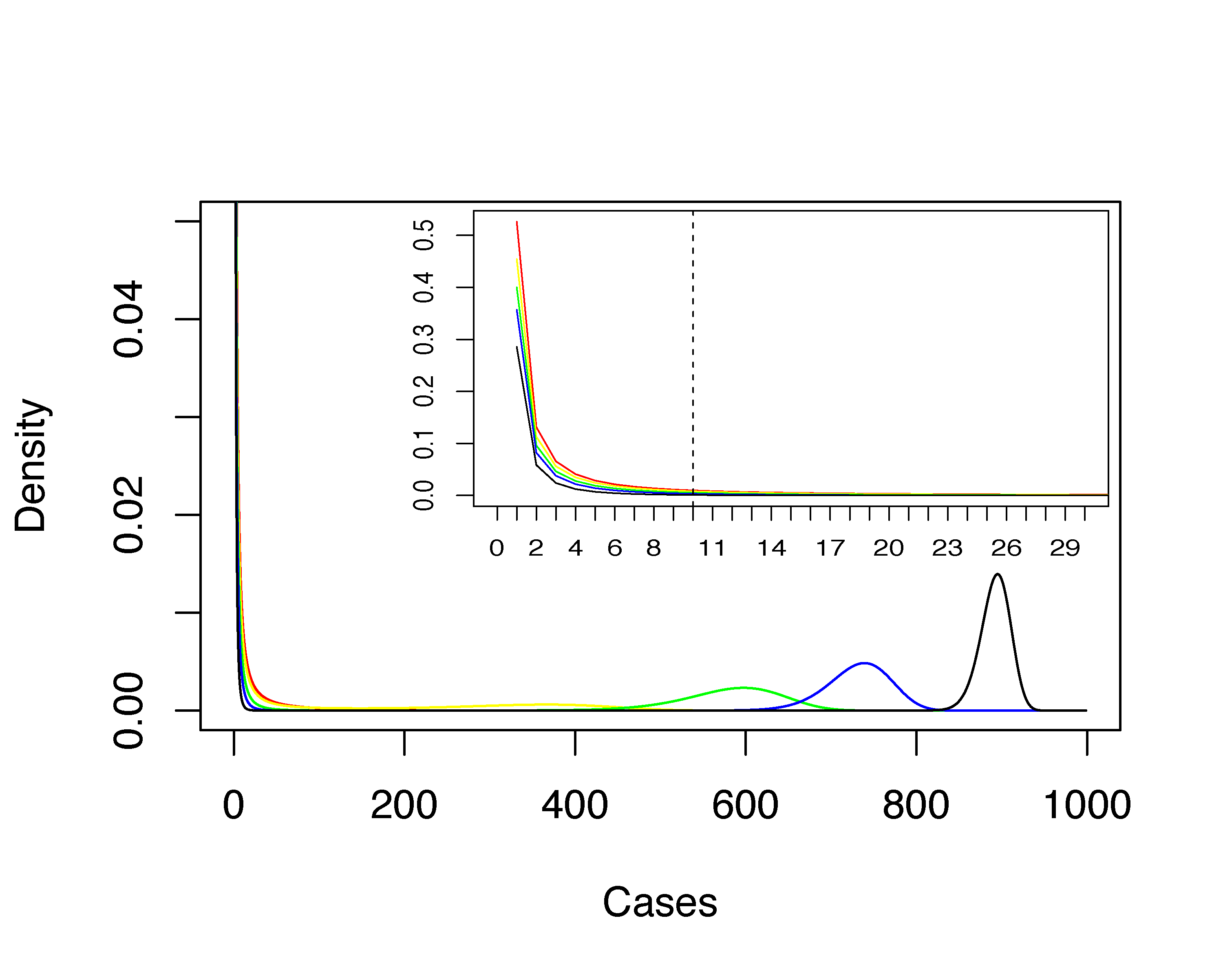

Supplement: S2 Fig — The final epidemic size distribution in a population of 1000 is monotonically decreasing when R0 equals 0.9 (red) and follows a bimodal distribution when R0 equals 1.2 (yellow), 1.5 (green), 1.8 (blue), and 2.5 (black). The inset shows a cutoff of 10 cases can discriminate large and small outbreaks with high sensitivity, but specificity can be low with low values of R0. (TIF) [file pntd.0006257.s006.tif]

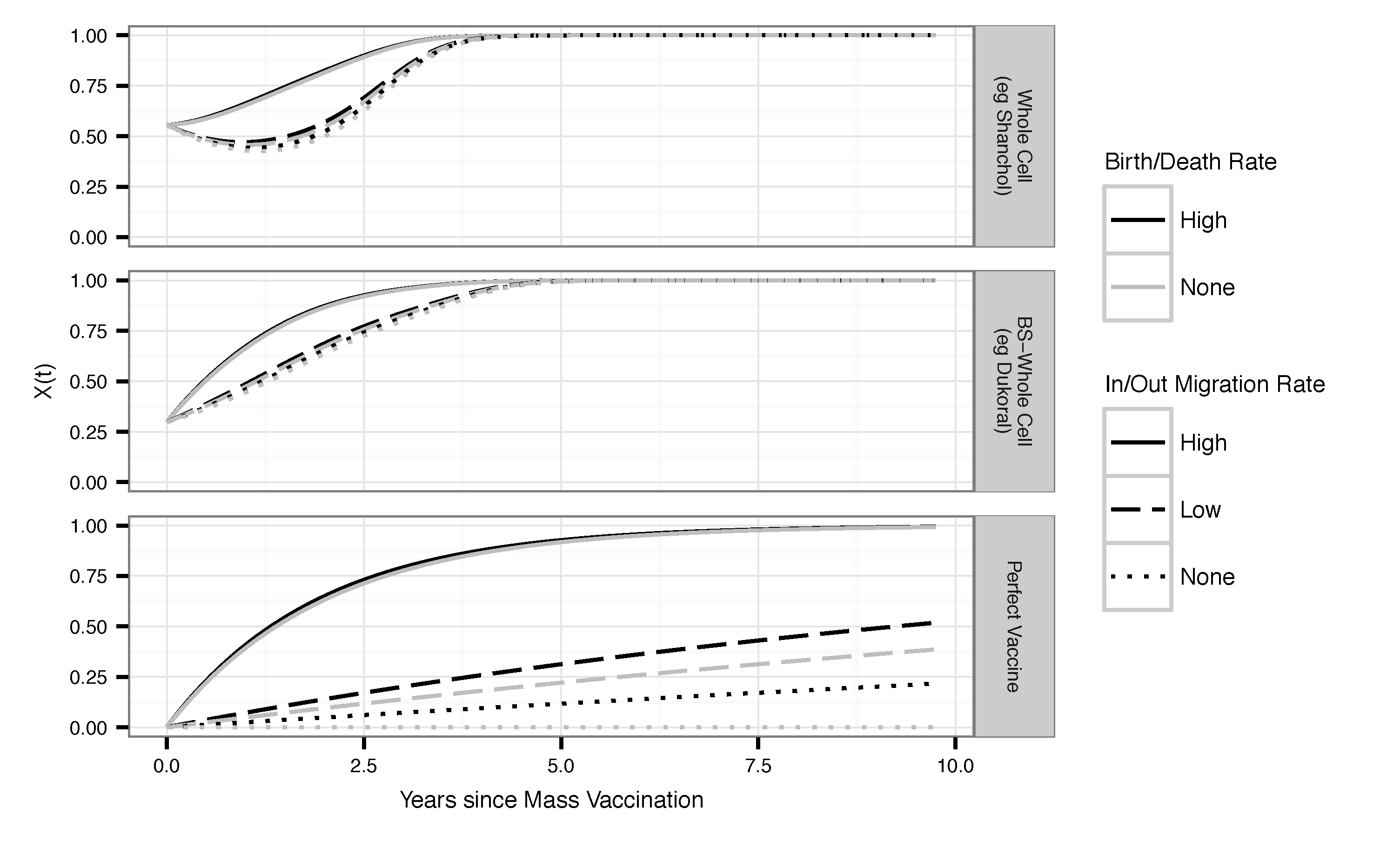

Supplement: S3 Fig — As per Fig 2A and 2B, but with the addition of high birth/death rates (140years) and the Whole Cell vaccine profile (without the B-subunit). Even conservatively fast rates of birth and death (140years) are slow compared to the rates of vaccine efficacy waning and high (12years) or low (120years) migration, and therefore have little impact. Note that linetype (i.e., solid, dashed, and dotted) apply to birth/death rates both high (black) and low (grey); therefore, dashed grey lines refer to simulations with no demographic turnover and low migration rates. (TIF) [file pntd.0006257.s007.tif]

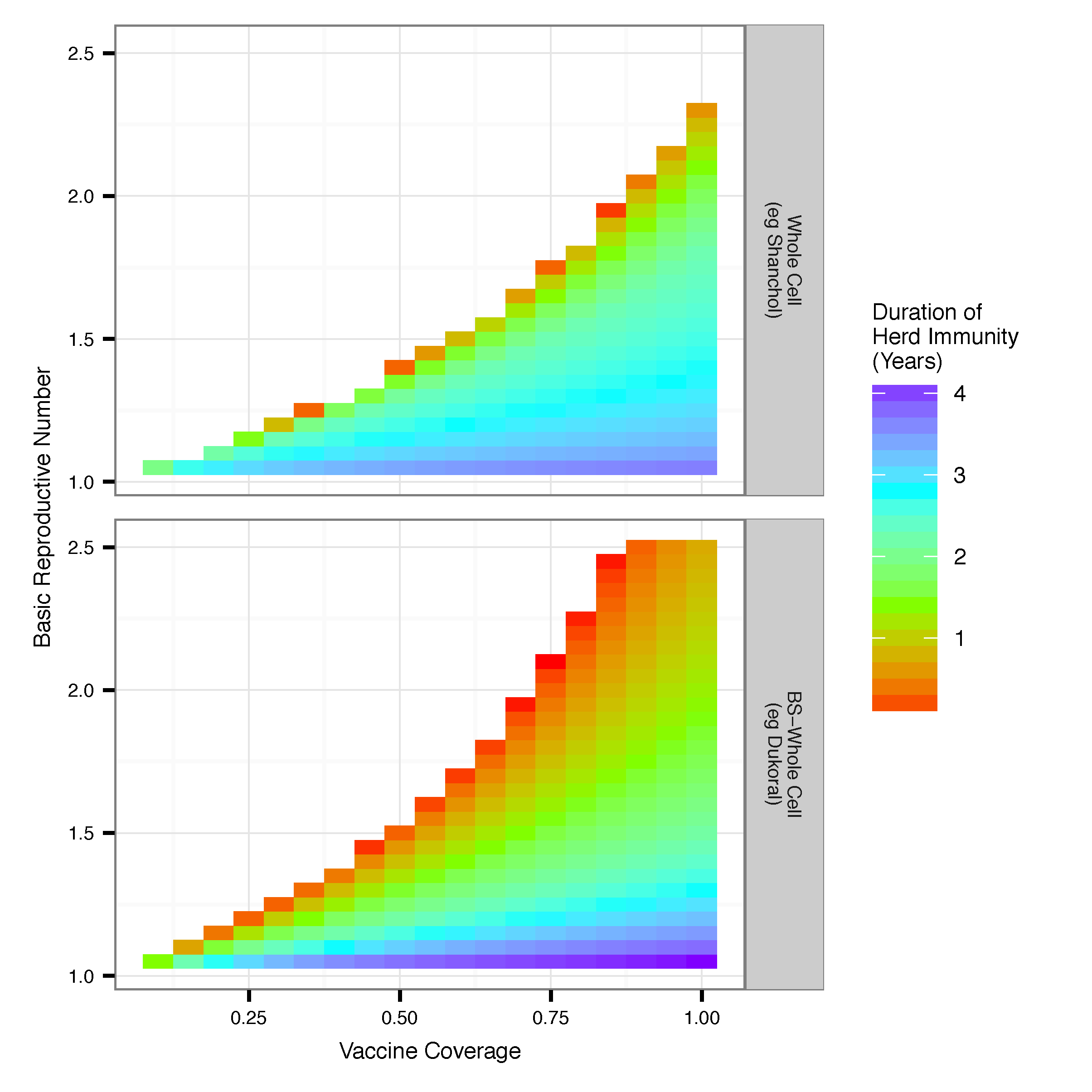

Supplement: S4 Fig — For both the whole cell and whole cell (with B-subunit) kOCVs, DHI is maximized in settings with high vaccine coverage and low basic reproductive numbers. Migration rates are set to zero. Uncolored regions never obtain herd immunity. (TIF) [file pntd.0006257.s008.tif]

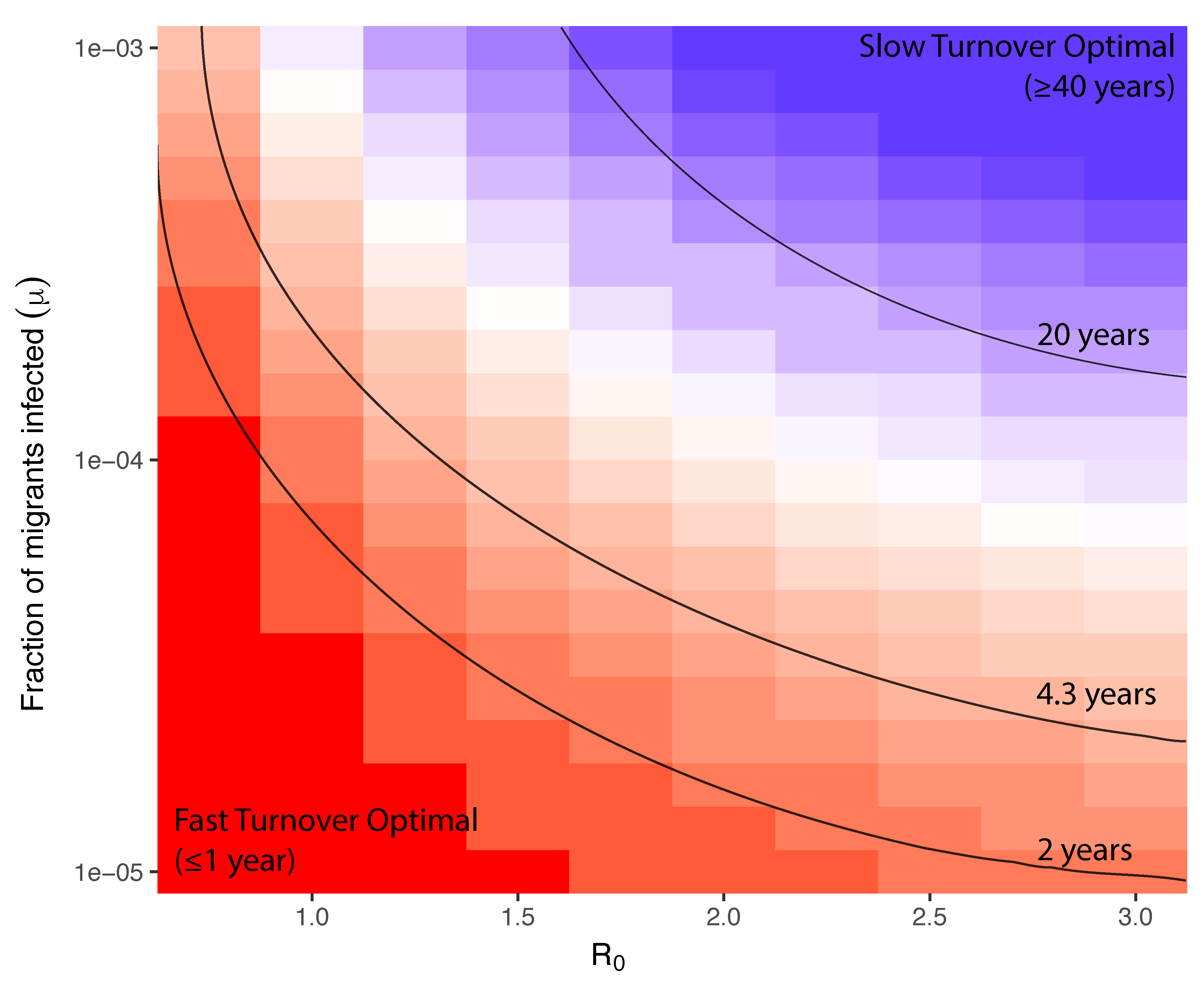

Supplement: S5 Fig — With increases in R0 or the fraction of migrants infected, μ, the optimal migration rate decreases from the fastest tested rate, 11year (red), to the slowest tested rate, 140years (blue). Contour lines denote average residence time in years from case studies in Dhaka (2), Bentiu (4.3), and Calcutta (20). For each simulation, the population size, N, is set to 10,000. (TIF) [file pntd.0006257.s009.tif]

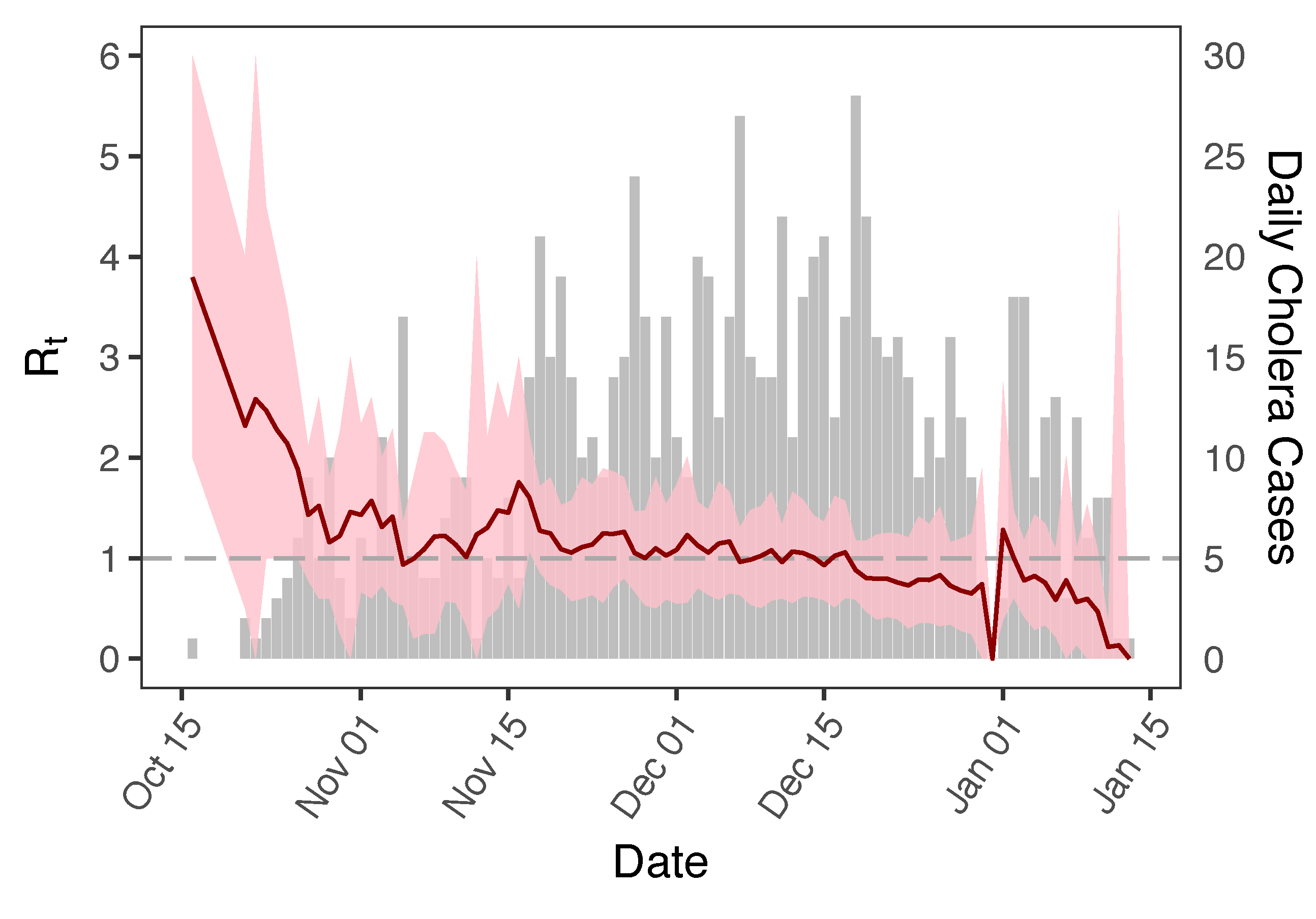

Supplement: S6 Fig — Using the daily case counts (grey bars) and a generation interval with median of 5 days and following a gamma distribution with shape = 0.5 and rate = 0.1 as per ref [37], we report a mean time-dependent reproductive number (red line) above unity for nearly two months. 95% confidence intervals are shown in pink. (TIF) [file pntd.0006257.s010.tif]

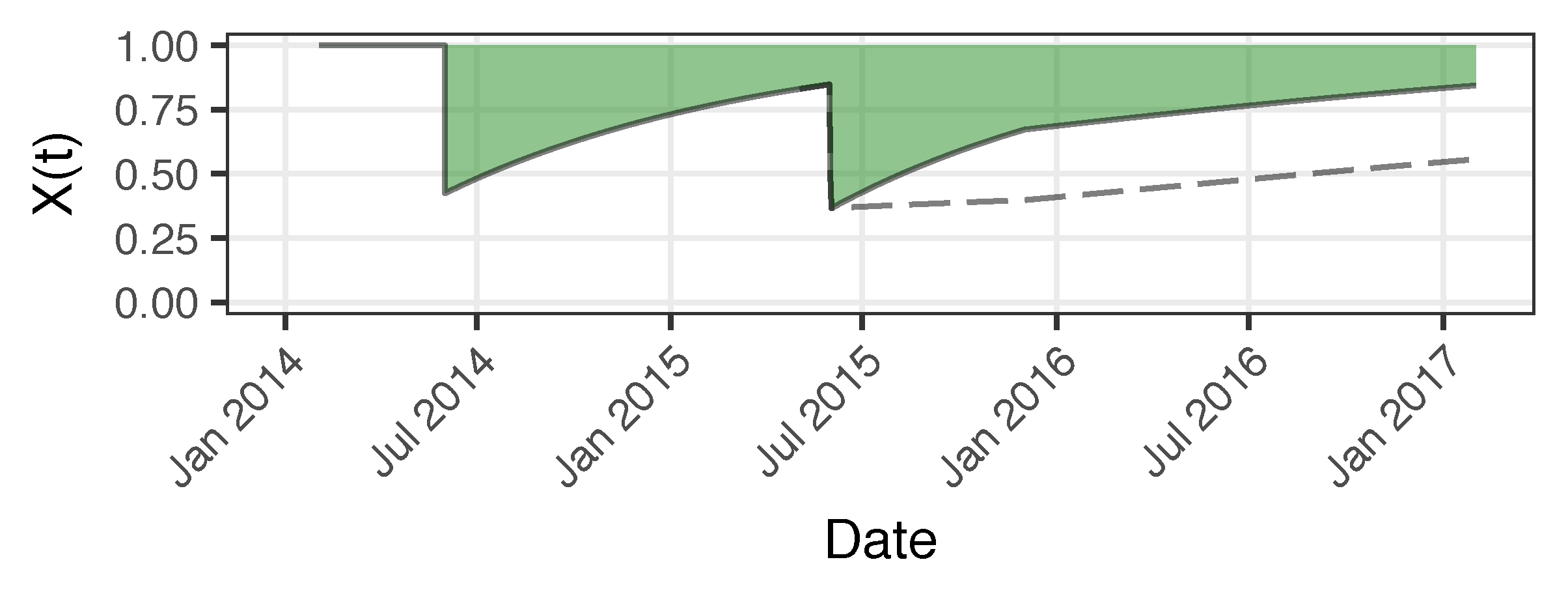

Supplement: S7 Fig — As per Fig 5B, with an additional dashed line indicating a counterfactual scenario whereby vaccines were administered to 100% of the estimated 55,628 new entries to the camp after the second mass vaccination campaign. With this strategy, population susceptibility on October 16, 2016 is 0.52 (dashed line), as compared to 0.81 in the absence of the Mass and Maintain strategy (solid green line). (TIF) [file pntd.0006257.s011.tif]

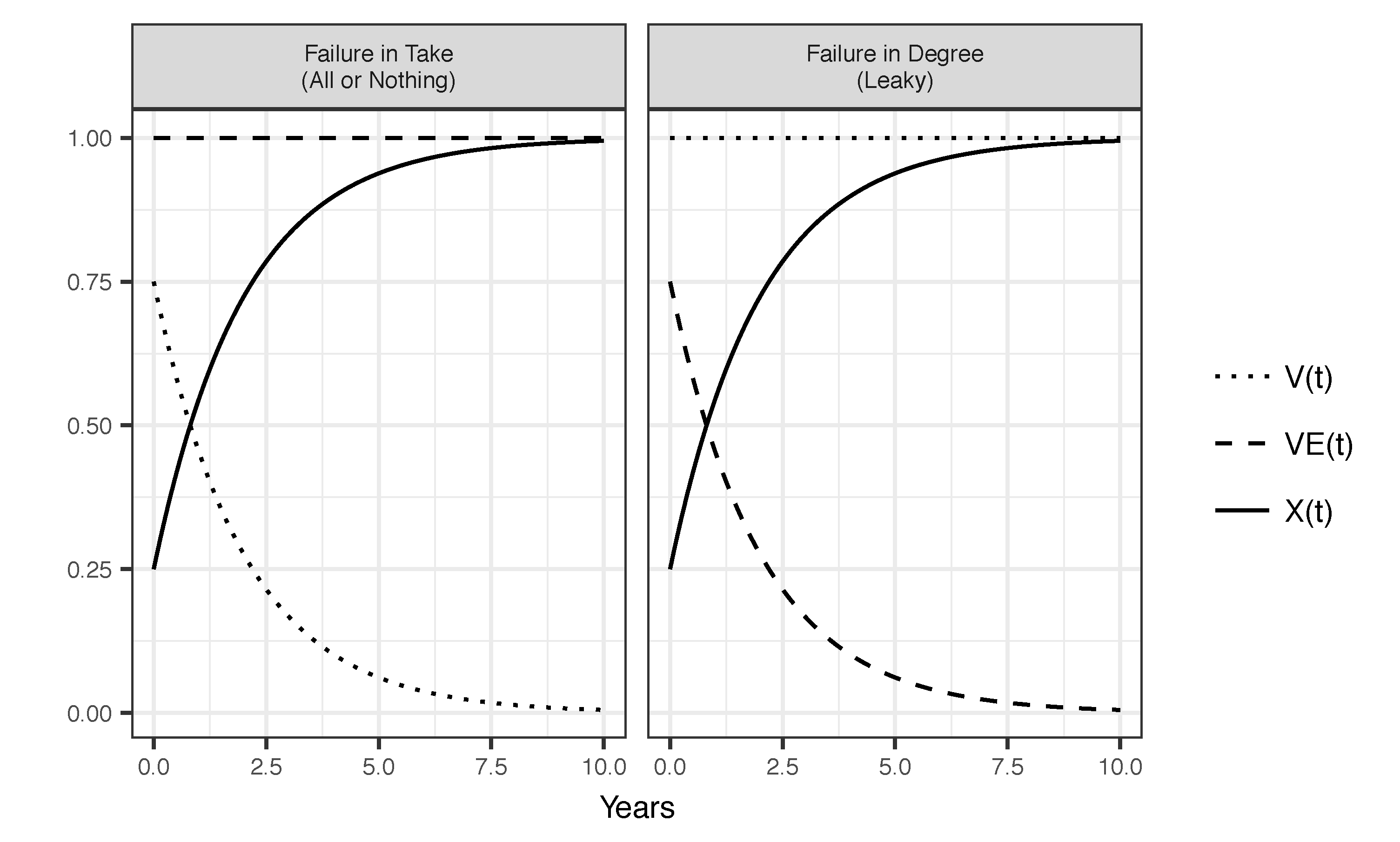

Supplement: S8 Fig — Vaccine efficacy waning that is due to a time-dependent failure in “take” (i.e., an “All or Nothing” vaccine waning) (left panel) retains a constant VE(t) (dashed lines) while the number of individuals in the V(t) ensemble decreases over time (dotted lines) from 75% to 0% using a theoretical example vaccine. For a time-dependent failure in “degree (i.e., a leaky vaccine waning) (right panel), individuals remain in the V(t) ensemble, but vaccine efficacy wanes from 75% to 0%. The proportion susceptible over time, X(t), calculated by Eq 5 is identical for both modes of action (solid lines). (TIF) [file pntd.0006257.s012.tif]

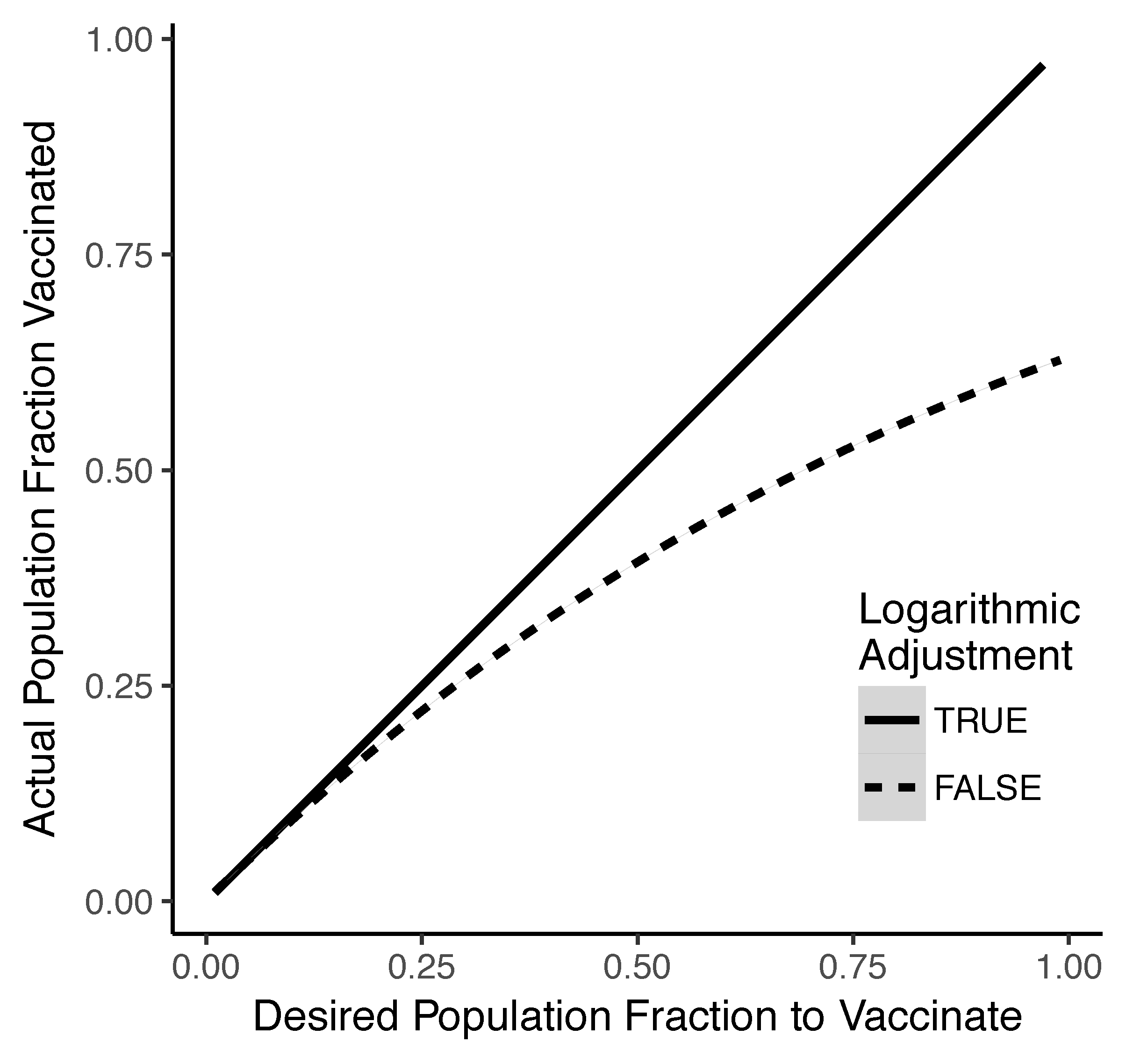

Supplement: S9 Fig — As the desired fraction of individuals to be vaccinated in a single day increases (x axis), the vaccination transition rate with the logarithmic adjustment (see supplementary materials) moves the accurate fraction of the population into the V1 compartment (solid line) while a transition rate that is simply equal to just the number of vaccines to be used (dashed line) does not move enough individuals into V1. (TIF) [file pntd.0006257.s013.tif]
